# Phytoplankton Succession in Recurrently Fluctuating Environments

**DOI:** 10.1371/journal.pone.0121392

**Published:** 2015-03-24

**Authors:** Daniel L. Roelke, Sofie Spatharis

**Affiliations:** 1 Texas A&M University, Department of Wildlife and Fisheries Sciences, and Department of Oceanography, 2258 TAMUS, College Station, Texas, United States of America; 2 University of Glasgow, Institute of Biodiversity, Animal Health and Comparative Medicine, Glasgow, Scotland, United Kingdom; University of Sydney, AUSTRALIA

## Abstract

Coastal marine systems are affected by seasonal variations in biogeochemical and physical processes, sometimes leading to alternating periods of reproductive growth limitation within an annual cycle. Transitions between these periods can be sudden or gradual. Human activities, such as reservoir construction and interbasin water transfers, influence these processes and can affect the type of transition between resource loading conditions. How such human activities might influence phytoplankton succession is largely unknown. Here, we employ a multispecies, multi-nutrient model to explore how nutrient loading switching mode might affect phytoplankton succession. The model is based on the Monod-relationship, predicting an instantaneous reproductive growth rate from ambient inorganic nutrient concentrations whereas the limiting nutrient at any given time was determined by Liebig’s Law of the Minimum. When these relationships are combined with population loss factors, such as hydraulic displacement of cells associated with inflows, a characterization of a species’ niche can be achieved through application of the R* conceptual model, thus enabling an ecological interpretation of modeling results. We found that the mode of reversal in resource supply concentrations had a profound effect. When resource supply reversals were sudden, as expected in systems influenced by pulsed inflows or wind-driven mixing events, phytoplankton were characterized by alternating succession dynamics, a phenomenon documented in inland water bodies of temperate latitudes. When resource supply reversals were gradual, as expected in systems influenced by seasonally developing wet and dry seasons, or annually occurring periods of upwelling, phytoplankton dynamics were characterized by mirror-image succession patterns. This phenomenon has not been reported previously in plankton systems but has been observed in some terrestrial plant systems. These findings suggest that a transition from alternating to “mirror-image” succession patterns might arise with continued coastal zone development, with crucial implications for ecosystems dependent on time-sensitive processes, e.g., spawning events and migration patterns.

## Introduction

Coastal lagoons, estuaries and bays are among the most productive systems on the planet and they provide essential habitat for many of the world’s species [1]. Much of this productivity and diversity is linked to phytoplankton productivity and assemblage composition. The temporal turnover in phytoplankton composition, or succession, in these marine systems is influenced by a complex interplay among many factors that sometimes include temperature and stratification, inorganic nutrient and light availability, zooplankton grazing and allelopathy [2–4]. Thus, factors acting at the base of the food web, such as these, can ultimately influence ecosystem form and functioning.

In many marine systems, variations within an annual cycle in freshwater inflows and mixing with the ocean lead to alternating periods of reproductive growth limitation involving multiple nutrients [5–10]. In turn, shifts in nutrient availabilities can influence succession patterns in phytoplankton. The literature on this phenomenon is well developed for inland water bodies, with field observations interpreted through in-field experimentation, laboratory monoculture work, and conceptual and numerical modeling [11–14]. Some notable succession patterns involve shifts away from diatom dominance towards dominance of green algae, as silica availability relative to other nutrients decreases and while nitrogen to phosphorus ratio is high. Further succession from this assemblage composition can involve shifts from green algae towards nitrogen-fixing cyanobacteria, as nitrogen availability relative to phosphorus decreases (see syntheses and reviews in [15, 16]). Similar observations have been made in marine systems [17–20], and recent work has focused on linking phytoplankton life history traits to succession patterns [21].

Marine systems where phytoplankton succession is sensitive to multiple inorganic nutrients likely differ in the mode of switching between limiting nutrients. For example, sudden changes in nutrient availabilities might arise in systems where inflow transitions are abrupt, e.g. following short-period runoff events associated with precipitation [22–25], or short-period vertical mixing events associated with winds [26–28]. On the other hand, gradual changes in nutrient availabilities might arise in systems that are characteristic of protracted wet and dry seasons, or annually occurring periods of upwelling [29, 30]. Gradual changes in nutrient availabilities might also arise in systems where the watershed supplying inflows contains reservoirs. During reservoir operations peak in-stream flows tend to be lower relative to historical (pre-dam) conditions and low in-stream flows tend to be elevated, resulting in a less dynamic flow regime [31, 32]. In addition, watersheds receiving interbasin water transfers during times of low in-stream flow may also become less dynamic.

In this research, we employ a multispecies, multi-nutrient mathematical model to explore how nutrient loading switching mode, i.e., sudden vs. gradual, might effect succession within phytoplankton assemblages. Such information will increase our understanding of how phytoplankton systems might respond to increased landscape alterations driven by human development and population growth. To keep our model tractable, we focus on succession patterns influenced solely by competition for reproductive growth limiting resources. Other succession-influencing factors, such as temperature, stratification, light, grazing and allelopathy, are not explored here.

## Methods

Our multispecies, multi-nutrient model was governed by the widely-used Monod-relationship, which predicts an instantaneous reproductive growth rate from ambient inorganic nutrient concentrations [33]. We used Liebig’s Law of the Minimum to determine which inorganic nutrient was limiting to each species at any point during model simulations [34]. As we will describe in the sections below, we combined these relationships with a population loss factor, i.e., hydraulic displacement of cells associated with inflows, to characterize each species’ niche using the R* conceptual model [11], thus enabling an ecological interpretation of our modeling results.

Our model simulations involved phytoplankton assemblages that self-organized from an initial species-rich pool [35]. Which species survived the self-organization process was determined by their competitive abilities (R* values) for two resources. Here, the half-saturation coefficients for resource-limited reproductive growth (*k*_*S*_, see below) and the fixed cellular content of resources (*Q*_*S*_, see below) were the life history traits that differentiated competing species. The numerical procedures we followed to establish the fluctuation modes in the resource supply, i.e., sudden and gradual transitions, are described below. Once self-organized phytoplankton assemblages emerged under these fluctuation modes, we compared the recurrent patterns of succession. Because we employed a degree of stochasticity to both the phytoplankton life history traits and the resource supply fluctuations, we reported on the emergent model behavior of 100 simulations for each of the resource supply fluctuation modes. Richness data from these simulations is provided in the supplementary material (S1 Data).

### Mathematical model and numerical solution

We employed a well-known mathematical model for depicting population dynamics of primary producers, i.e., plants, macroalgae and phytoplankton, that is governed by the Monod-relationship [11, 13, 36]. We structured the model to simulate a phytoplankton assemblage where new resources arrived with inflow, and loss of cells and ambient nutrients occurred through hydraulic flushing. As mentioned previously, for this research we intentionally kept the model structure simple so that interpretation of results was more tractable. The compounding influence of other abiotic and biotic factors, such as temperature, stratification, light, grazing and allelopathy, coupled with resource supply fluctuation mode on phytoplankton succession patterns was beyond the scope of this research.

For each species, population dynamics were simulated using an equation of the form:

$$\frac{dN}{dt}=\mu N-\nu N$$ (1)

where *N* was population density (cells liter^-1^), μ was specific reproductive growth rate (d^-1^), and ν was hydraulic flushing defined as the inflow divided by the system volume (d^-1^). For the purposes of this research, we assume that carbon content per cell was constant, so *N* became an analog of biomass.

Specific reproductive growth rate for each population was determined using the Monod equation [33] and Liebig's "Law of the Minimum" [34] following the form:

$$\mu =\mu_{\text{max}}(\text{min}[\frac{S1}{S1+k_{S1}},\frac{S2}{S2+k_{S2}}])$$ (2)

where *μ*_*max*_ was the maximum specific reproductive growth rate for a species (d^−1^), *S1* and *S2* were the concentration of resources necessary for reproductive growth (μM), and *k*_*S1*_ and *k*_*S2*_ were the half-saturation coefficients for resource-limited reproductive growth (μM) specific to each species. A function ‘min’ was used to determine which resource was more strongly limiting to reproductive growth for a given species at each time step of the simulation. Parameterizations of *μ*_*max*_ and *k*_*S1*_ for each species, and in regards to *S1*, were based on a range of literature values reported for nitrogen. The parameterizations of *k*_*S2*_ for each species and *S2* were based on nitrogen-equivalents, an approach used previously for ease of programming multi-nutrient models and analyzing results [37, 38].

For the two resources necessary for reproductive growth, changes in concentration were simulated using an equation of the form:

$$\frac{dS}{dt}=\nu (S_{source}-S)-\sum_{i=1}^{n}Q_{S_{i}}\mu_{i}N_{i}$$ (3)

where *S*_*source*_ was the fixed concentration of the resource in the source (μM), *Q*_*S*_*i*__ was the fixed cellular content of the resource (μmole cell^−1^) for a species, *n* was the total number of species, and other parameters were the same as previously described.

Differential equations were solved numerically using ordinary differential equation solving routines that were a part of a commercial software package (The Math Works, Inc.). The routines were based on fourth-order Runge-Kutta procedures, and used a variable time step that was based on a local error tolerance set at 10^−15^.

### Fluctuations in the resource supply

Assemblages self-organized under an annually fluctuating resource supply (*S*_*source*_). Simulations were initiated with the first resource having a concentration of 2 μM and the second resource a concentration of 20 μM which are within realistic ranges of resource concentrations in river discharges into coastal lagoons, estuaries and bays where anthropogenic eutrophication is not as prevalent [39]. Over a period of ∼182 days, the concentrations of the resources in the supply reversed. That is, at day 182 the first resource had a concentration of 20 μM and the second resource a concentration of 2 μM. This annually recurring nutrient reversal periodicity was selected as it is the most representative of natural systems [40, 41].

**Fig 1:**
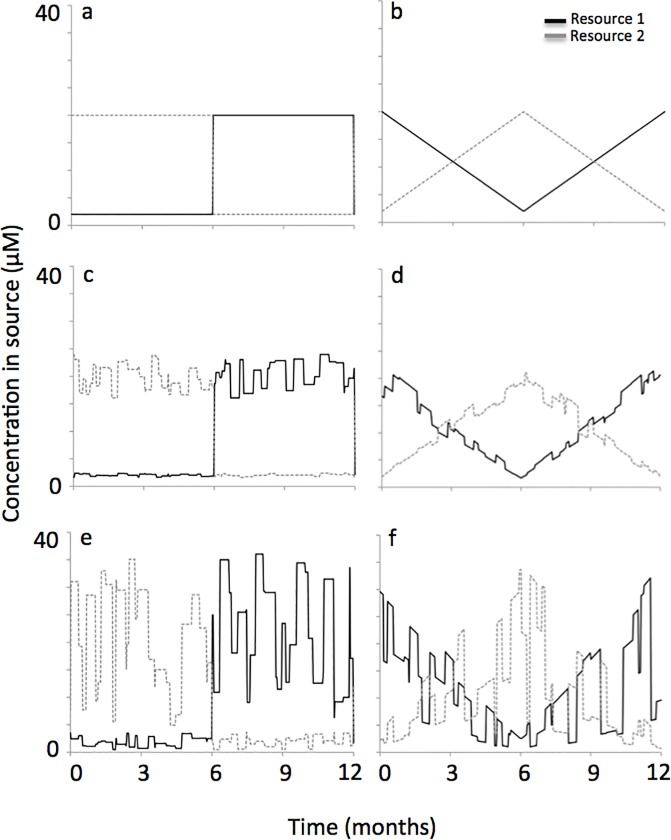
Sudden and gradual modes of reversal in the resource supply concentration. The sudden mode of reversal went from a resource supply of 2 and 20 μM for resources 1 and 2 to a resource supply of 20 and 2 μM with an abrupt transition on day 182. Three levels of noise in the resource supply were explored, which were no noise (a), 0–20% noise (c) and 0–80% noise (e). The gradual mode of reversal also went from a resource supply of 2 and 20 μM for resources 1 and 2 to a resource supply of 20 and 2 μM, but with a slow change that reversed direction on day 182. Again, three levels of noise in the resource supply were explore, which were no noise (b), 0–20% noise (d) and 0–80% noise (f).

We explored two modes of this resource supply reversal. The first mode was a sudden change, where resource supply concentrations were 2 and 20 μM for days 1 to 182, then abruptly switched to 20 and 2 μM on day 182 and remained at those concentrations through day 365. On day 365, they abruptly switched back to concentrations of 2 and 20 μM (see Fig. 1A). The second mode of resource supply reversal was a gradual change, where the first resource progressively increased from 2 μM on day 1 to 20 μM on day 182, while the concentration of the second resource progressively decreased from 20 μM on day 1 to 2 μM on day 182. From days 182 through 365 the resources progressively changed back to their initial values (see Fig. 1B). Thus, the fluctuation period for both modes of resource supply reversal occurred over an interval of 365 days. As mentioned previously, switching between limiting resources over a period of a year is observed in many natural systems, especially when the year is characterized by wet and dry seasons [5–10].

In natural settings, however, it is unlikely that resource supply reversals are quite so regular. Consequently, we preformed additional simulations with added noise to the resource supply. We created a function that randomly altered the resource concentration in the supply by adding or subtracting a value equivalent to some percentage of the resource supply concentration that was used for the ‘regular’ scenarios (described in the preceding paragraph and shown in Fig. 1A, B). The function also accounted for the duration that the resource supply was altered. More specifically, the adjusted resource concentration in the supply was randomly varied in magnitude over the interval [0–20%] (see Fig. 1C, D for representative simulations) or over the interval [0–80%] (see Fig. 1E, F for representative simulations). This resource supply adjustment, or amount of noise in the resource supply concentration, occurred over periods that randomly varied in duration from 1 to 14 days. We selected these intervals because they generated simulation results for resource supply variability similar to what is observed in multiple coastal systems [18, 42, 43].

### Parameterization of populations to produce self-organized, species-rich assemblages

We generated phytoplankton assemblages using a numerical approach that involved self-organization from a species-rich pool. The number of species in the initial pool was 300. The authors have worked in a variety of aquatic ecosystem types that include lakes, rivers, bays and coastal oceans [18, 44–46]. Field sampling in these systems usually spanned multiple seasons, with sampling frequencies ranging from weekly to quarterly. Phytoplankton species richness in these ecosystems ranged from one to ∼80, with 15–20 species being the norm. Thus, we considered the initial species pool of 300 used in these simulations as “species rich”. The simulated period of self-organization was 15 years. Surviving species after this simulated period (those with biomass >0.05% of the total biomass) were then used to initiate a second 15-year simulation. The surviving species from this second simulation were considered members of a self-organized assemblage. In this way, we assured ourselves that the processes underlying coexistence in our simulations followed from life history traits and not potential effects from an initial species-rich pool.

Parameterization of life history traits, i.e., reproductive growth-related factors in our simulations, was not random. We used existing knowledge of physiological traits and basic ecological principles as a guide. More specifically, the resource-specific, half-saturation coefficient for reproductive growth (*k*_*S*_) and the cellular resource content (*Q*_*S*_) varied proportionally and this trade-off was demonstrated previously [38]. To illustrate, *k*_*S*_ was selected randomly from numbers within the range 0.04–1 which when expressed in units of μM represents the range typically observed for half-saturation coefficients in phytoplankton [14, 47]. Following the principal of proportionality mentioned above, *Q*_*S*_ was then set equal to *k*_*S*_. When in units of 10^−6^ μmole cell^−1^ the range of 0.04–1 represents typical resource contents measured in phytoplankton [14, 47]. Therefore, species with relatively low *k*_*S*_ and *Q*_*S*_ in our simulations represented populations with high competitive ability for that resource and low demand. Conversely, species with relatively high *k*_*S*_ and *Q*_*S*_ represented populations with low competitive ability for that resource and high demand. This type of inverse relationship was observed in phytoplankton cultures for some resources, such as silica, but not for other resources, such as phosphorus [38].

Our parameterization of reproductive growth-related factors was further guided by a trade-off between competitive abilities for the two resources used in the model. A species being a good competitor for one resource meant that it was a poor competitor for the other resource; and a species being an intermediate competitor for one resource meant that it was also an intermediate competitor for the other resource [11, 48]. This prevented any single species from being a superior competitor for both resources. To illustrate, a species with *k*_*S1*_ and *Q*_*S1*_ values of 0.15 for resource 1 would then have *k*_*S2*_ and *Q*_*S2*_ values of 0.85 for resource 2. This species would be characterized as a good competitor for resource 1 and a poor competitor for resource 2. A species with *k*_*S1*_ and *Q*_*S1*_ values of 0.45 for resource 1 would then have *k*_*S2*_ and *Q*_*S2*_ values of 0.55 for resource 2. This species would be characterized as an intermediate competitor for resources 1 and 2. These life history traits were further modified to target an intermediate level of complementarity in the initial species pool [49], which was observed previously in phytoplankton assemblages [38].

### R* model framework

To provide a mechanistic understanding of the effect of nutrient loadings on species with multiple competitive abilities and their succession patterns we employed the R* conceptual model [11]. As mentioned above, this model depicts niche spaces for multiple populations along a resource trade-off line. One can visualize how an ecosystem characteristic of recurrent nutrient fluctuations can be described by nutrient shifts back and forth along the resource trade-off line. As this happens, each population experiences a period when it is the superior competitor, i.e., the part of the resource trade-off line near the population’s optimal resource ratio. Because the system keeps moving along the resource trade-off line, competitive exclusion is much more limited compared to conditions where the resource supply is constant [12]. This enables many species to coexist. Higher diversity of life history traits can be visualized when species’ optimal resource ratios are distributed over greater lengths of the resource trade-off line.

The competitive ability of each species for each resource (the R*) was determined based on one of their life-history traits, *k*_*S*_ (recall that the other life history trait specific among species, *Q*_*S*_, was proportional to *k*_*S*_). Along with *μ*_*max*_ and *v*, which were the same for all species in the assemblage, knowledge of *k*_*S*_ enabled determination of R* values for each species [11, 16]. This was achieved following the equation:

$$R^{*}=\frac{\nu k_{S}}{\mu_{\text{max}}-\nu }$$ (4)

**Fig 2:**
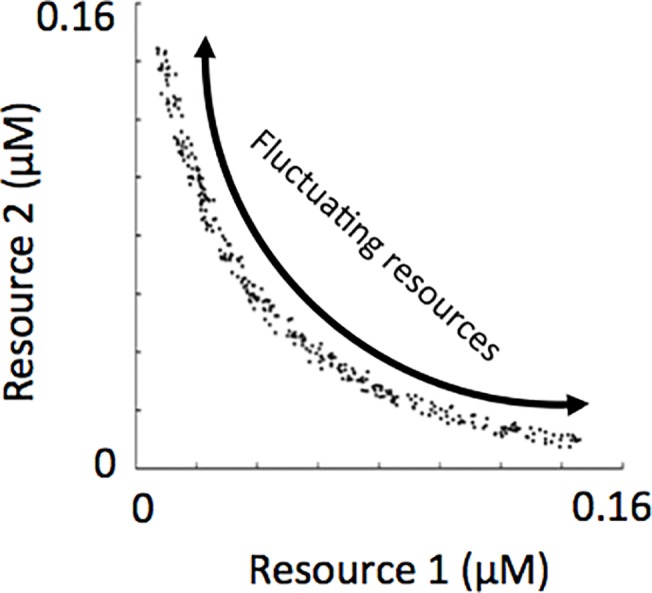
R*s of phytoplankton in the initial species pool and the recurrently fluctuating environmental conditions from which assemblages self-organized.

Our simulations employed two resources therefore the resource trade-off space was two-dimensional. As mentioned previously, we employed a resource trade-off curve characteristic of intermediate complementarity [38]. Consequently, the distribution of species’ R*s through a two-dimensional resource trade-off space was downward curved (see Fig. 2). The modes of resource supply fluctuation described in Fig. 1 can also be envisioned by following a trajectory along the resource trade-off curve, as depicted in Fig. 2.

### Adding noise to the life-history traits

Prior to the simulations mentioned above, a low amount of noise was added to each life history trait not exceeding 0.04. This is why the species’ R*s do not line up exactly along the resource trade-off curves shown in Fig. 2. In a natural system, it is not likely that R*s would line up exactly on a line through the resource trade-off space. Adding some noise to *k*_*S*_ and *Q*_*S*_ added realism to the model. In this research, we did not explore the relationship between the amount of noise added to the life history traits and the assemblage characteristics of interest.

### Model initialization

Initial population densities for each species were the same for all simulations, i.e., *N* = 0.1 (x10^6^ cells liter^−1^). Initial resource concentrations were 2 μM for resource 1 and 20 μM for resource 2. Parameter constants included total flushing and maximum specific reproductive growth rate, which were *v* = 0.25 d^−1^ and *μ*_*max*_ = 2 d^−1^. As mentioned previously, values for *k*_*S*_ where within the range 0.04 and 1.0 (μM), and values for *Q*_*S*_ ranged within 0.04 and 1.0 (10^−6^ μmole cell^−1^). Values of *k*_*S*_ and *Q*_*S*_ for some of the representative assemblages shown in our results are reported in Tables 1, 2 and 3. For all simulations, all parameterizations were within the range of what is typical for phytoplankton assemblages and pelagic environments [14, 47].

**Table 1:** Life history traits (*k*_*s*_ and *Q*_*s*_ for resources S1 and S2) of species from representative simulations shown in Fig. 4.

	Resource fluctuation was sudden (Fig. 4A)				Resource fluctuation was gradual (Fig. 4B)			
	*k*_*s*_		*Q*_*s*_		*k*_*s*_		*Q*_*s*_	
Species	S1	S2	S1	S2	S1	S2	S1	S2
1	0.3350	0.3123	0.3664	0.3335	0.8037	0.0842	0.8132	0.0845
2	0.8619	0.0741	0.8834	0.1086	0.5619	0.1619	0.5636	0.2017
3	0.6757	0.1216	0.6843	0.1418	0.9869	0.0468	0.9870	0.0471
4	1.0001	0.0458	1.0184	0.0810	0.4141	0.2563	0.4169	0.2743
5	0.7836	0.0960	0.8138	0.0989	0.7045	0.1119	0.7232	0.1459
6	0.0639	0.9078	0.0982	0.9109	0.2280	0.4461	0.2303	0.4748
7	0.0425	1.0334	0.0535	1.0392	0.0813	0.8082	0.1200	0.8083
8	0.2523	0.4112	0.2560	0.4370	0.3188	0.3286	0.3521	0.3443
9	0.7804	0.0983	0.8125	0.0998	0.0484	0.9900	0.0591	1.0048
10	0.9462	0.0556	0.9634	0.0608	0.0470	1.0034	0.0789	1.0425
11	0.0475	1.0033	0.0522	1.0203	0.4363	0.2367	0.4708	0.2663
12	0.0520	0.9801	0.0650	0.9989	0.2226	0.4496	0.2473	0.4826
13	0.1918	0.5087	0.2275	0.5320	0.0461	1.0032	0.0475	1.0140
14	0.1559	0.5835	0.1680	0.5950	0.4461	0.2277	0.4557	0.2480
15					0.4171	0.2530	0.4234	0.2830

**Table 2:** Life history traits (*k*_*s*_ and *Q*_*s*_ for resources S1 and S2) of species from representative simulations shown in Fig. 6.

	Resource fluctuation was sudden (Fig. 6A)				Resource fluctuation was gradual (Fig. 6B)			
	*k*_*s*_		*Q*_*s*_		*k*_*s*_		*Q*_*s*_	
Species	S1	S2	S1	S2	S1	S2	S1	S2
1	0.7679	0.0921	0.7927	0.1217	0.3968	0.2625	0.4117	0.2730
2	0.3288	0.3160	0.3358	0.3395	0.4931	0.2028	0.4989	0.2396
3	0.2543	0.4032	0.2805	0.4189	0.7333	0.1023	0.7652	0.1367
4	0.7818	0.0902	0.8034	0.0936	0.9109	0.0576	0.9227	0.0641
5	0.9891	0.0490	1.0095	0.0747	0.6562	0.1301	0.6673	0.1560
6	0.1350	0.6298	0.1615	0.6667	0.5667	0.1658	0.5722	0.1876
7	0.0524	0.9674	0.0746	0.9973	0.3255	0.3222	0.3265	0.3545
8	0.7790	0.0914	0.7989	0.1285	0.1509	0.5878	0.1590	0.6131
9	0.1264	0.6544	0.1375	0.6919	0.0447	0.9945	0.0520	1.0268
10	1.0087	0.0453	1.0229	0.0502	0.3630	0.2905	0.3929	0.3116
11	0.4358	0.2369	0.4556	0.2505	0.0609	0.9090	0.0979	0.9310
12					0.0652	0.8802	0.0843	0.8887
13					0.1835	0.5237	0.2059	0.5454
14					0.1758	0.5350	0.1922	0.5380

**Table 3:** Life history traits (*k*_*s*_ and *Q*_*s*_ for resources S1 and S2) of species from representative simulations shown in Fig. 7.

	Resource fluctuation was sudden (Fig. 7A)				Resource fluctuation was gradual (Fig. 7B)			
	*k*_*s*_		*Q*_*s*_		*k*_*s*_		*Q*_*s*_	
Species	S1	S2	S1	S2	S1	S2	S1	S2
1	0.4799	0.2106	0.5126	0.2435	0.3088	0.3402	0.3270	0.3624
2	0.3065	0.3457	0.3077	0.3703	1.0064	0.0484	1.0191	0.0757
3	1.0022	0.0442	1.0355	0.0581	0.4352	0.2313	0.4524	0.2388
4	0.0491	0.9771	0.0803	0.9928	0.5734	0.1579	0.5745	0.1835
5	0.0477	0.9990	0.0655	1.0232	0.8603	0.0728	0.8684	0.0802
6	0.3617	0.2923	0.3769	0.2984	0.1998	0.4997	0.2270	0.5114
7	0.3688	0.2861	0.3693	0.3134	0.1441	0.6185	0.1491	0.6291
8					0.0487	0.9817	0.0823	1.0114
9					0.8264	0.0791	0.8399	0.0910
10					0.1047	0.7247	0.1223	0.7314

## Results

**Fig 3:**
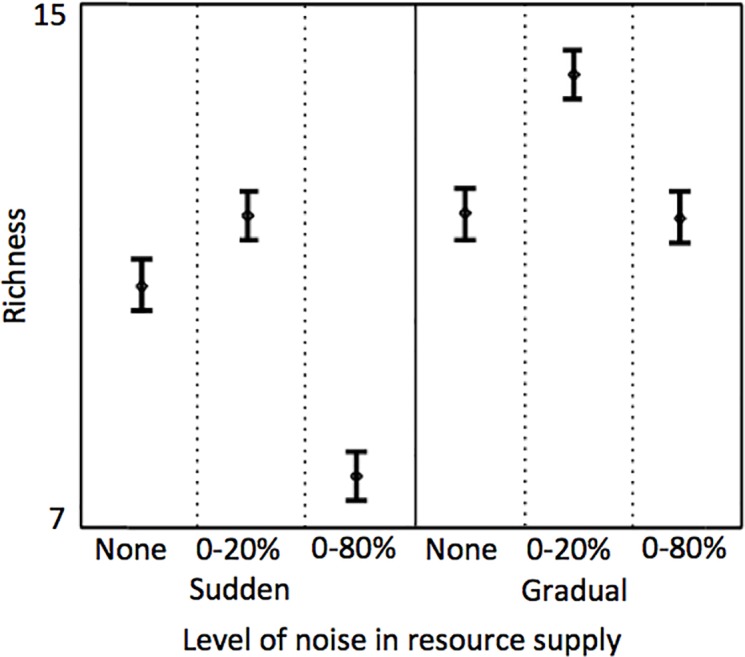
Averaged species richness (bars are 95% confidence interval) in the self-organized phytoplankton assemblages (*n* = 100) for the scenarios where reversals in the resource supply concentrations were sudden (left) and gradual (right), each at the three levels of noise in the resource supply. Data for richness data are provided as supplementary material (S1 Data).

In all simulations (600 total), species-rich assemblages emerged from the process of self-organization. Here, species rich refers to a condition where the number of coexisting populations was greater than the number of limiting resources (i.e. >2). This occurred regardless of the mode of reversal in the resource supply concentrations, and regardless of the amount of noise applied to the resource supply concentrations. In both modes of reversal in the resource supply, richness was greatest at the intermediate noise level, i.e. 0–20% with richness being greater in scenarios where resource supply reversals were gradual compared to scenarios where resource supply reversals were sudden (Fig. 3).

**Fig 4:**
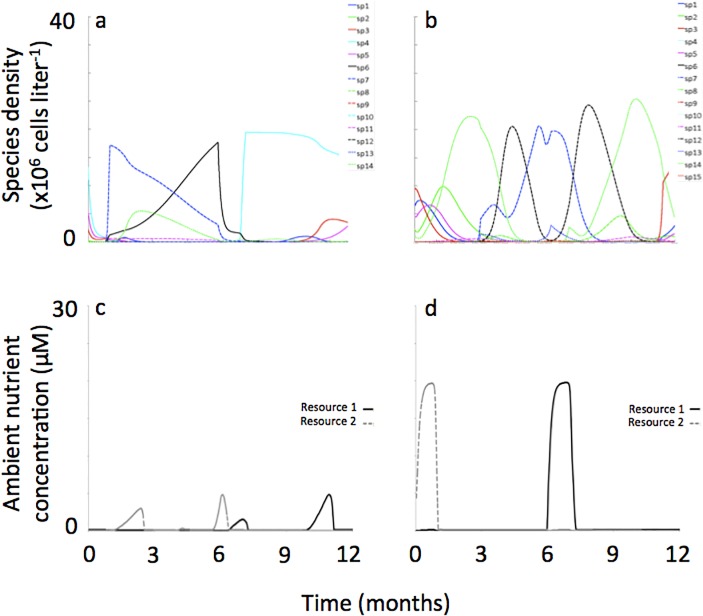
Representative phytoplankton population dynamics and ambient resource concentrations when reversals in the resource supply concentrations were sudden (a, c) and gradual (b, d). In these simulations there was no noise applied to the resource supply. Values of *k*_*S*_ and *Q*_*S*_ for phytoplankton populations are provided in Table 1.

The modes of resource supply reversal produced distinct patterns of recurrent population dynamics. When resource supply reversals were sudden, back-to-back patterns of population dynamics resulted. We illustrate this below using a sequence of numbers, where each number represents a species that was dominant or subdominant, and the sequence of numbers represents the change in dominance over time. For example, following the representative simulation shown in Fig. 4A, where ‘numbers’ correspond to species (see figure panel legend), the sequence of species succession was: **[**7 (6,11→14,6,1,11) → 6 (7,14,11)**]** → **[**4 (14→1→3→5)**]** where ‘number’ sequences shown within **[]’**s indicate species succession during a period when one resource was supplied at a lower concentration than the other, ‘number’ sequences shown within ()’s indicate succession among subdominant species, and the **]** → **[**notation indicates the point during succession where supply of resources switched from one resource being supplied at greater concentration to the other resource being supplied at greater concentration.

**Fig 5:**
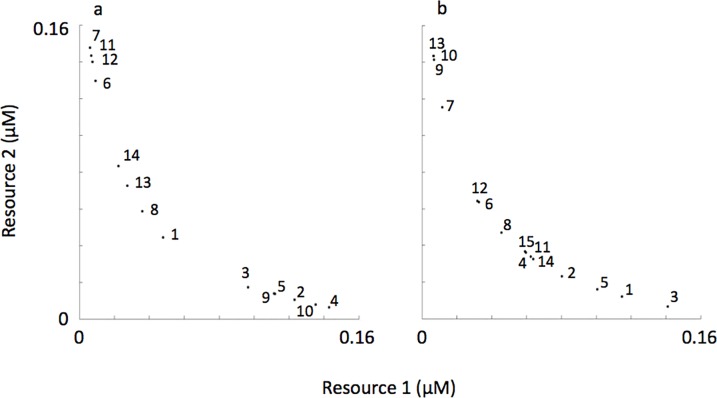
R*s of representative self-organized phytoplankton assemblages shown in Fig. 4 where reversals in the resource supply concentrations were sudden (a) and gradual (b). The numbers on the figure panels coincide with the species keys shown in Fig. 4A, B.

These alternating patterns, when coarsely reflected upon, show succession dynamics among species situated at either extreme of the resource trade-off curve (Fig. 5A). That is, when resource 1 was being supplied at a lower concentration, species 7 and 6, both with low R*s for resource 1, were the dominant species at that time in the succession sequence. When resource 2 was being supplied at a lower concentration, species 4, with the lowest R* for resource 2, was the dominant species at that time in the succession sequence.

**Fig 6:**
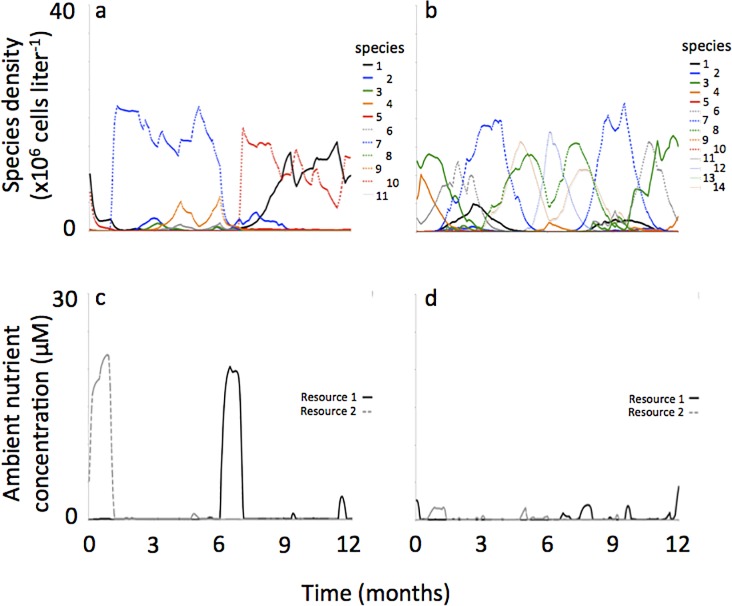
Representative phytoplankton population dynamics and ambient resource concentrations when reversals in the resource supply concentrations were sudden (a, c) and gradual (b, d). In these simulations there was 0–20% noise applied to the resource supply. Values of *k*_*S*_ and *Q*_*S*_ for phytoplankton populations are provided in Table 2.

**Fig 7:**
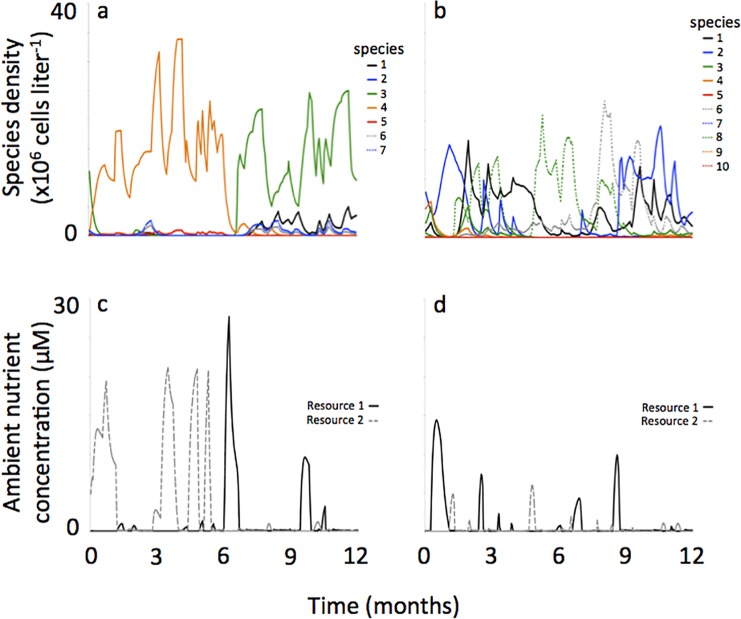
Representative phytoplankton population dynamics and ambient resource concentrations when reversals in the resource supply concentrations were sudden (a, c) and gradual (b, d). In these simulations there was 0–80% noise applied to the resource supply. Values of *k*_*S*_ and *Q*_*S*_ for phytoplankton populations are provided in Table 3.

But a closer examination of the succession pattern in Fig. 4A shows a sequence among subdominant species characteristic of low R*’s for one of the resources (i.e., species 11, 1 and 3) and species of intermediate R*’s (i.e., species 1 and 14) (Fig. 5B). And the succession sequence among these species did not follow the R* ranking shown on the resource trade-off curve. In addition, some species never came to dominate, or even to become sub-dominant, despite having very low R*’s for one of the resources (i.e., species 12, 2, 9 and 10). These subtle and complex alternating succession patterns occurred regardless of the amount of noise in the resource supply concentrations (representative simulations shown in Figs. 6A, 7A).

When resource supply reversals were gradual, “mirror-image” population dynamics resulted (see Fig. 4B). Here, “mirror image” refers to a succession pattern observed while one resource was limiting that is reversed, i.e., occurring in the opposite sequence, when the other resource was limiting. For example, a succession from species A to species B, then to species C in reverse would be species C to B to A. So, the mirror-image recurrent pattern would then be A-B-C-B-A-B-C-B-A-, etc. Regarding the representative simulation shown in Fig. 4B, the succession pattern was: 3 → 1 → 5 → 2 → 14(11)**]** → **[**14(7,13) → 12(8) → 7(13) → 12(8)**]** → **[**14(8→11) where notations were the same as described previously. In this sequence, strong “mirror-image” population dynamics are seen following the sequence 3-14-12-7-12-14-3, etc.

Like simulations where resource supply transitions were sudden, in simulations were resource supply transitions were gradual succession dynamics were dominated by species situated at either extreme of the resource trade-off curve when corresponding resources were in short supply. For example, when resource 1 was being supplied at a lower concentration, species 7 and 12, both with low R*s for resource 1, were the dominant species in the succession sequence (Figs. 4B, 5B). When resource 2 was being supplied at a lower concentration, species 3, 1 and 5, all with low R*s for resource 2, were the dominant species.

But unlike simulations where resource supply transitions were sudden, here the sequence of species succession generally followed the R* ranking along the resource tradeoff curve. For example, in the representative simulation shown in Fig. 4B the species succession starts off with 3-1-5-2- as resources gradually shift from low concentration of resource 2 towards low concentration of resource 1. This was the same ranking in the R*s with species 3 having the lowest R* for resource 2 followed species 1-5-2- with R*s getting progressively higher (Fig. 5B). Also different from simulations where resource supply transitions were sudden, here the amount of noise in the resource supply concentrations influenced mirror-image succession. At higher amounts of noise, mirror-image succession was less obvious (Figs. 6B, 7B).

Regarding ambient resource concentrations, switches between which resource was more limiting to productivity within a half-year period sometimes occurred, where the incidence of these switches was a function of the amount of noise applied to the resource supply concentrations. For example, in the simulations where no noise was applied there were no switches between the two reproductive growth limiting resources within a half-year period (Fig. 4C, D). But in the simulations were 0–80% noise was applied to the resource supply there were multiple switchings between the two reproductive growth limiting resources within a half-year period (Fig. 7C, D). This relationship between switches in limiting resources and noise in the resource supply was observed for both scenarios of sudden and gradual reversals in resource supply concentrations.

## Discussion

Our simulation results showed sustained high species richness in gradually fluctuating environments, which is consistent with theory [11, 16] and many previous studies focused on phytoplankton [50–52]. Interestingly, in our scenarios where shifts in resource supply concentrations were sudden, i.e., where the period of unchanging external loading of resources was 180 days, we still observed high species richness. This is likely because the ambient resource concentrations continued to fluctuate even though the external supply was constant. These fluctuations were driven by species interactions through competition for resources, a type of intrinsic disturbance. This phenomenon was reported in previous models where resource supply was held constant [37, 53]. Those models, however, required a minimum of three resources in the supply for richness to be maintained. In a recurrently fluctuating system, such as the one modeled here, these intrinsic disturbances operated with only two resources in the supply. In coastal systems characteristic of multiple limiting resources [5–10], sudden transitions in resource supply concentrations often occur as a result of pulsed inflows or vertical mixing events driven by intermittent winds [23–28]. It is likely that in these systems, this mechanism of intrinsic disturbance brought on by phytoplankton competition of resources contributes to maintenance of high species richness.

Richness was slightly greater when fluctuations in the resource supply were gradual in our simulation results. This likely happened because more species characteristic of intermediate R*s are able to survive when there is a slower transition between the two limiting resources as in the case of the gradual change in resource supply concentrations. The application of noise in the resource supply enhanced species richness in both scenarios of sudden and gradual fluctuation, but only when the noise was not excessive. It is intuitive that noise in the resource supply would reduce the amount of time resources are at either extreme of the resource trade-off curve. In turn, this would enhance survivability of species characteristic of intermediate R*’s. However, when noise is further increased (0–80%) this seems to have a negative effect on species richness. This may be a result of resources dropping for significant periods of time (from 1 to 14 days) to levels lower than those required for species to grow (i.e. below the R*s) thus resulting in species extinctions.

Observations of alternating succession patterns in our model are consistent with some observations in natural environments, especially from inland water bodies. Recall that succession patterns among diatoms, and eventually green algae have been observed early in the season and represent a species succession distinct from that which occurs later in the season, often involving a shift from different green algae to multiple cyanobacteria species [15, 16]. The processes driving these seasonal successions are many and include temperature, light and nutrient limitation effects, accumulation of zooplankton populations and top-down control through less-selective grazing, grazer-driven nutrient regeneration, a clear water phase, and a second accumulation of zooplankton populations and partial top-down control through preferential grazing. Distinct patterns of seasonal phytoplankton succession such as these are likely to occur in coastal environments as well, as many of the mechanisms that drive these seasonal successions for inland water bodies also operate in coastal systems [2–4, 19]. Here, however, we show that alternating succession patterns can emerge solely by competition for resources, provided that the recurrent fluctuation in the resource supply is sudden. As mentioned previously, this would be the likely mode of resource supply reversal in systems characteristic of pulsed inflows and/or episodic vertical mixing [23–28].

We also observed “mirror-image” succession in our model, but to the best of our knowledge, this has never been observed in a real-world plankton environment. There is some evidence of mirror-image succession patterns in terrestrial environments. For example, in some mangrove plant communities some species have annual bimodal patterns of dominance in reproductive phenology where the peaks are separated by other species showing an annual unimodal pattern of dominance in reproductive phenology. These community flowering and fruiting patterns were linked to day-length, rainfall and temperature [54]. Similarly, some forests species of herbs showed an annual bimodal pattern in flowering phenology where the peaks were separated by species of shrubs and vines showing annual unimodal patterns in flowering phenology. These community patterns were driven by an interplay between annual changes in winds, and insect and bird pollinators [55]. Also, in some forest floor and grassland plant communities annual bimodal peaks in bryophyte production was separated by annual unimodal peaks in herbaceous plant production. These were driven by annual patterns in light, temperature and moisture [56]. In all of these examples from terrestrial environments, the forcing factors occurred gradually over the course of a year, an observation that our model predictions are consistent with. So, it may be that mirror-image succession patterns are not observed in phytoplankton systems because gradual changes in resource supply do not occur in those systems. Alternatively, it may be that resource supplies to aquatic systems are noisy, which our model suggests would prevent mirror-image succession patterns from emerging.

Some nuanced aspects of our results suggest limitations of R*model application to interpretation of succession patterns observed in recurrently fluctuating environments. But they also illuminate the complex relationship between resource loading and phytoplankton succession. For example, frequently in our simulations two or more species with very similar R*’s (driven by *k*_*S*_’s in our model) and *Q*_*S*_’s showed widely different patterns of dominance and population dynamics. Sometimes one species would dominate while another nearly identical species would show very little accumulation of biomass. In addition, sometimes one species would show a population dynamic where accumulation and decline in biomass occurred over a period of weeks to months while another nearly identical species would maintain accumulated biomass for several months. Finally, the sequence of species succession did not always follow the ranking of R*’s along the resource trade-off curve, especially in simulations where the resource supply reversal was sudden. These complex relationships between resource loading and phytoplankton succession underscore the importance of coupling conceptual and numerical models when interpreting real-world observations.

## Conclusions

In the research we show that the mode of reversal and the amount of noise in the resource supply strongly influence phytoplankton species richness and succession. Sudden reversals in resource supply concentrations led to alternating succession dynamics of slightly less species richness, while gradual reversals in resource supply concentrations led to mirror-image succession of slightly higher species richness. Our understanding of these relationships will better enable us to predict how phytoplankton composition and population dynamics in coastal lagoons, estuaries and bays might change as human populations and development in coastal zones increase, both of which may lead to future conditions more characteristic of gradually fluctuating resource supplies instead of sudden fluctuations. Such a shift might decrease the prevalence of alternating succession dynamics, possibly leading to a more erratic succession pattern when environmental noise is considered. In turn, this might disrupt time sensitive processes associated with other aspects of the food web, such as spawning and migration cycles. Finally, the plethora of population biomasses and dynamics that emerged within these self-organized assemblages further illuminated the complex relationship between resource loading and phytoplankton, underscoring the importance of coupling conceptual and numerical models when interpreting real-world observations.

## Supporting Information

S1 Data Supplementary material on richness data. One-hundred simulations were performed for each of the six scenarios where transitoons in the resource supply concentrations were either sudden or gradual, and the amount of noise in the resource supply concentrations was either none, 0–20% or 0–80%.
(PDF)
