# Supplementary material for: Phytoplankton Succession in Recurrently Fluctuating Environments
Source: PLoS One. 2015 Mar 24;10(3):e0121392. doi: 10.1371/journal.pone.0121392 (PMC4372531; doi:10.1371/journal.pone.0121392)
Supplement: S1 Data — One-hundred simulations were performed for each of the six scenarios where transitoons in the resource supply concentrations were either sudden or gradual, and the amount of noise in the resource supply concentrations was either none, 0–20% or 0–80%. (PDF) [file pone.0121392.s001.pdf]

S1 Richness data. One-hundred simulations were perfomred for each of the six scenarios where transitoons in the resource supply concentrations were either sudden or gradual, and the amount of noise in the resource supply concentrations was either none, 0-20% or 0-80%.

	Sudden Transitions			Gradual Transitions		
simulation	No Noise	0-20% Noise	0-80% Noise	No Noise	0-20% Noise	0-80% Noise
1	13	12	6	9	14	8
2	6	9	4	12	12	12
3	11	13	4	12	20	15
4	10	13	7	10	11	17
5	12	11	5	10	9	14
6	19	16	10	11	15	12
7	8	8	6	11	18	12
8	9	17	5	14	16	9
9	6	8	7	15	12	15
10	13	12	9	14	15	6
11	14	9	8	11	12	12
12	9	9	9	15	8	12
13	12	10	11	11	10	16
14	11	6	9	8	14	9
15	13	4	4	11	15	13
16	12	10	5	12	12	13
17	12	4	6	13	11	9
18	14	10	4	16	10	14
19	8	11	6	9	16	8
20	10	20	8	11	16	16
21	7	6	8	9	11	14
22	14	10	7	8	14	12
23	13	10	8	14	12	13
24	5	17	8	14	17	11
25	12	15	10	9	11	9
26	12	9	9	14	10	8
27	12	10	10	11	10	16
28	12	13	6	8	13	7
29	11	12	12	20	16	14
30	10	10	5	10	17	10
31	11	17	8	16	15	10
32	11	12	7	13	13	9
33	9	11	8	19	16	13
34	9	7	9	16	13	11
35	8	9	9	8	12	12
36	11	12	5	13	17	10

37	7	14	7	13	12	15
38	18	16	10	10	16	12
39	14	13	7	12	14	12
40	12	13	10	15	10	13
41	10	14	4	9	16	12
42	12	8	9	9	14	11
43	8	14	6	12	10	11
44	12	12	6	14	12	14
45	7	13	6	12	15	12
46	4	9	13	10	17	9
47	9	10	5	8	18	16
48	11	10	10	9	14	14
49	15	14	7	11	13	13
50	6	10	7	11	18	11
51	12	10	7	14	8	13
52	6	10	4	13	10	15
53	6	11	7	14	18	11
54	8	12	3	17	17	11
55	19	12	8	12	13	7
56	10	9	7	14	17	14
57	9	19	10	11	15	9
58	16	15	14	13	13	16
59	8	11	8	6	13	11
60	10	13	7	15	13	12
61	6	9	5	15	17	13
62	13	12	3	14	9	15
63	16	10	6	9	23	7
64	7	11	8	13	16	12
65	10	9	14	12	15	10
66	10	12	8	12	17	14
67	13	16	8	14	16	12
68	11	12	6	10	12	8
69	11	16	5	15	11	13
70	17	13	6	14	12	12
71	8	13	7	8	14	11
72	12	14	13	9	18	9
73	13	12	3	10	15	10
74	13	12	8	15	15	14
75	9	10	5	9	13	11
76	12	10	9	15	12	13
77	10	9	7	9	19	14
78	12	15	14	17	12	12

79	6	13	6	12	13	11
80	5	14	5	15	15	8
81	12	12	10	11	15	13
82	8	13	5	8	13	12
83	12	12	13	11	14	12
84	7	11	11	9	17	12
85	11	12	6	10	19	10
86	15	9	15	8	14	12
87	13	13	5	14	20	15
88	18	11	14	12	16	18
89	11	9	10	12	20	13
90	7	16	10	8	13	10
91	11	14	12	11	16	9
92	17	8	6	7	14	9
93	13	14	9	10	11	11
94	13	22	7	10	16	11
95	6	12	6	11	12	14
96	11	12	2	16	11	11
97	6	21	9	8	12	8
98	7	8	7	18	15	15
99	10	17	8	9	15	11
100	9	9	5	13	13	8
